# Exploring Advanced Functionalities of Carbon Fiber-Graded PEEK Composites as Bone Fixation Plates Using Finite Element Analysis

**DOI:** 10.3390/ma17020414

**Published:** 2024-01-14

**Authors:** Chenggong Zhang, Pihua Wen, Yigeng Xu, Zengxiang Fu, Guogang Ren

**Affiliations:** 1 School of Engineering and Materials Science, Queen Mary University of London, London E1 4NS, UK; chenggong.zhang@qmul.ac.uk; 2 Institute of Aeronautics and Astronautics, School of Infrastructure Engineering, Nanchang University, Nanchang 330031, China; 3 School of Aerospace, Transport and Manufacturing, Cranfield University, Cranfield MK43 0AL, UK; yigeng.xu@cranfield.ac.uk; 4 Faculty of Life Science, Northwestern Polytechnical University, Xi’an 710072, China; fuzengx@nwpu.edu.cn; 5 School of Physics, Engineering and Computer Science, University of Hertfordshire, Hatfield AL10 9AB, UK

**Keywords:** functionally graded materials, finite element analysis, fixation plate, stress shielding, CCF/PEEK

## Abstract

This study aims to address the challenges associated with conventional metallic bone fixation plates in biomechanical applications, such as stainless steel and titanium alloys, including stress shielding, allergic reactions, corrosion resistance, and interference with medical imaging. The use of materials with a low elastic modulus is regarded as an effective approach to overcome these problems. In this study, the impact of different types of chopped carbon fiber-reinforced polyether ether ketone (CCF/PEEK) functionally graded material (FGM) bone plates on stress shielding under static and instantaneous dynamic loading was explored using finite element analysis (FEA). The FGM bone plate models were established using ABAQUS and the user’s subroutine USDFLD and VUSDFLD, and each model was established with an equivalent overall elastic modulus and distinctive distributions. The results revealed that all FGM bone plates exhibited lower stress shielding effects compared to metal bone plates. Particularly, the FGM plate with an elastic modulus gradually increased from the centre to both sides and provided maximum stress stimulation and the most uniform stress distribution within the fractured area. These findings offer crucial insights for designing implantable medical devices that possess enhanced mechanical adaptability.

## 1. Introduction

A bone is made of dynamic and living material whose structure and density continuously evolve and develop in response to biological and mechanical environments [1,2]. It is a smart self-generating material under stress during the human lifecycle, has the ability to change its architecture by the removal of old bones and replacing them with newly formed bone structures in a localized biological process [3,4]. There are two patterns of the bone fracture healing process: primary healing and secondary healing [5,6,7]. For primary healing to occur, it is essential to achieve a precise anatomical realignment of the fractured ends, ensuring no gaps and maintaining stable and direct contact. However, such ideal conditions are seldom found in the natural healing process [6,7]. In most cases, the contact is unstable and indirect, and fractured bones will be healed by secondary healing, which involves a combination of intramembranous and endochondral ossification [8]. In secondary healing, when the bone healing process begins, mechanical stimuli, such as stress and strain, play an important role [8]. Without enough surrounding stress as the natural stimulus, the restored bone would be brittle and loose, and may be refractured prematurely after bone plate removals [9,10].

Metallic bone fixation plates are commonly used to help restore bones in orthopaedic applications, especially the fixation of long bone fractures [11,12]. Upon initial implantation, an inflammatory response occurs at the implantation site, ultimately leading to the formation of a hematoma [13]. As the healing progresses, both soft callus and hard callus are successively formed [14]. The stability and stress stimulation provided by the implant are important for the formation of the callus [15]. Appropriate stress stimulation can activate chondrogenic cells [16,17] and bone precursor cells, which can respond to a mechanical stimulus [18], while excessive movement may disrupt the newly formed callus. Although metals are known for their high strength, ductility, and resistance to wear, they have the shortcomings of low biocompatibility, different levels of corrosion under human biological conditions, higher thermal conductivity, and magnetic and electrical field sensitivity [19,20]. One of the biggest problems has been that alloy, such as Ti_6_Al_4_V, possess an elastic modulus five times more than human cortical bones [21,22]. The mismatch between the modulus of the metallic plates and the cortical bones leads to a common scenario in which most of the loads are transferred through the plates rather than the underlying bones or bone structures [8,23,24,25]. This phenomenon is widely recognized as the “stress-shielding effect” or “stress-protection”, which delays or distorts the bone’s natural healing process [21,26,27].

Changing the geometry design of the bone fixation plate is a common method to reduce the stress shielding effect. Combining topology optimization with additive manufacturing, the equivalent stiffness of the metallic part can be adjusted, and the consumption of the materials can be reduced during the fabrication process [21,22]. However, these metallic implants left in the human body can lead to long-term problems, such as the release of metal ions, inflammatory reactions, and risk of infection [22].

The ideal solution is to produce a plate with controlled degradation of the modulus. At different healing stages, bone fixation plates can behave differently to avoid the stress shielding effect [28,29,30,31]. Biodegradable materials such as polylactic acid, polyglycolic acid, and their co-polymers can be degraded through the procedure of desertification. The degradation products of these polymers are lactic and glycolic acids, which could be safely absorbed or derived by body metabolism [29,32]. Although polylactic acid is utilized in various fields of bone repair, there are several pressing issues that require resolution. These issues include high production costs, reduced biocompatibility stemming from inert hydrophobic surfaces, uncontrollable degradation rates, the formation of acidic degradation by-products, and the need for enhanced antimicrobial properties [33].

One practical method for solving the stress shielding effect is to find alternative materials for the fixation plates where the requirement must be that the modulus of the fixation materials is as close as possible to the bone modulus. Polyether ether ketone (PEEK) is a high-performance thermoplastic polymer first commercially marketed as a long-term implanted biomaterial in April 1998 (Invibio Ltd., Thorton-Cleveland, UK) [34]. In addition to its superior mechanical properties suitable for biomedical applications, it gives good biocompatibility, low- and high-temperature durability, excellent wear and fatigue properties, and performance stability for enduring sterilization without degradation on both physical and chemical performance in vivo [34,35,36].

In clinical application, it is found that carbon fibre-reinforced PEEK (CF/PEEK) plates are radiolucent and, therefore, do not hinder radiographic evaluation, producing CT images with much fewer artifacts than any other implant fixation plates made of metal alloys [8,36]. Thus, it is convenient and beneficial in detecting the levels of healing with restored fractured bones in clinical applications. Moreover, the release of metal ions in metallic implants may cause allergic tissue reactions, which means that substituting metal with CF/PEEK can avoid potential hypersensitivity to metal implants [35,37]. Plates made of functionally graded materials (FGMs) were introduced to achieve a varying elastic modulus while maintaining the mechanical properties of the bone fixation plates. FGMs have a continuous spatial distribution of two or more components through the thickness of the product [38,39]. Short fibre-reinforced PEEK is an attractive functionally graded material for bone fixation plates due to its potential for rapid and low-cost mass production and its controllable elasticity modulus with the volume fraction of carbon fibre. The properties of spatially graded composites were altered by changing the volume fraction of carbon fibre.

In the present study, finite element analysis (FEA) was employed to conduct a comparative analysis regarding the stress shielding effects between FGM bone plates, fixed-content carbon fibre-reinforced PEEK bone plates, and conventional plates made of stainless steel and titanium alloys. In this work the bone plates with the least stress shielding effect were analysed, optimised, and concluded that functionally graded materials (FGMs) are better options suitable for internal bone fixation applications.

## 2. Materials and Methods

### 2.1. Model Geometry

**Figure 1:**
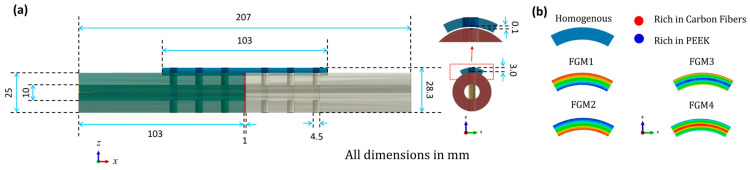
Geometry information of the 3D fractured bone/plate model: (**a**) detailed geometries of the 3D fractured bone/plate model; (**b**) schematic of the four types of FGM bone plates and homogeneous plates.

To optimize the elastic modulus of bone fixation plates in real experiments, 3D FEA model simulations were utilized to compare the mechanical performance of the CCF/PEEK plate with other material bone plates. Solidworks 2019 software was used to create 3D models, as shown in Figure 1a, while finite element analysis was conducted using ABAQUS 2020 software. The tibia was modeled as a hollow cylinder for simplification based on previous studies [10,40]. The geometric model was pre-set as follows:

(a) Tibia bone: A smooth, hollow cylinder with an inner diameter of 10.0 mm, an outer diameter of 25.0 mm, and a length of 103.0 mm.
(b) Fractured bone site: Set to 1 mm and match the outer and inner diameters of the tibia bone.
(c) Plates: Modelled with six holes, with holes drilled through both the bone plates and the bone thickness. The plate dimensions were 103.0 mm in length, 15 mm in width, and 3.0 mm in thickness.
(d) Screws: Represented as cylindrical cortical screws, fixed to the bone. The screws were simplified and had a diameter of Ø4.5 mm and a length of 28.3 mm.

### 2.2. Material Properties

The mechanical properties of healthy bones are taken as anisotropic, while the carbon fibre-reinforced hydroxyl apatite (CF/HA) bone plates are taken as transversely isotropic. Three healing stages of fracture fragments (1%, 50%, and 75%) were considered for the investigation. The callus is assumed to the bridge the fractured site, and the callus material is assumed to be isotropic and homogeneous [27,41]. The homogeneous chopped carbon fiber-reinforced PEEK (CCF/PEEK), stainless steel (ST316L), Ti6A14V (TC4), CF/HA, and four types of FGM plates were evaluated using the FEA model.

The bone, as a biological material, undergoes changes in density due to factors such as age, gender, physique, and pathological conditions. In this study, bone density data were derived based on the correlation between bone density and elasticity established in previous studies [42,43,44]. The correlation between apparent bone density and the elastic modulus is shown in Equation (1).

(1) $$E=3.891\rho_{app}^{2.39}$$

where *E* is the elastic modulus in GPa and *ρ_app_* is the apparent density in g/cm^3^.

The mechanical properties of all the materials used in the modelling analysis are collectively shown in Table 1 [10,23,27,45,46,47,48,49].

The average elastic modulus for all FGM bone plates is set as 22,530 MPa, which is the same as the homogeneous CCF/PEEK bone plates. A schematic of the four types of FGM bone plates and homogeneous plates is shown in Figure 1b. The FGM bone plates were labeled FGM1, FGM2, FGM3, and FGM4 based on the variation of mechanical properties.

Variations in the carbon fiber volume fraction result in a changing elastic modulus, Poisson’s ratio, and density along the thickness direction. FGM1 shows an increasing carbon fiber content from the bone proximate side to the bone distal side. FGM2 exhibits a decreasing carbon fiber content from the bone proximate side to the bone distal side. FGM3 displays a reduction in carbon fiber content from both sides of the bone plate toward the center. Conversely, FGM4 demonstrates an increase in carbon fiber content from both sides of the bone plate toward the center. The rule of mixture [50,51] was adopted to calculate the elastic modulus, Poisson’s ratio, and density of the FGM plates, as shown in Equation (2):

(2) $$P_{c}=V_{f}P_{f}+V_{m}P_{m}$$

where *P_c_* denotes a certain property of the composite, such as the elastic modulus, Poisson’s ratio, and density; and *V_f_* and *V_m_* denote the fiber and matrix volume fractions, respectively.

**Figure 2:**
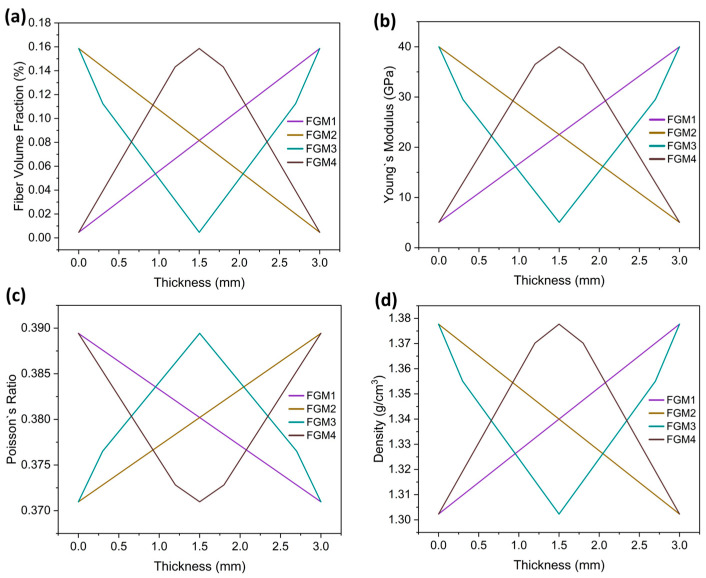
The material properties of the FGM plates: (**a**) gradient patterns of the fiber volume fraction of the four types of bone plates (**b**) gradient patterns of Young’s modulus of the four types of bone plates; (**c**) gradient patterns of Poisson’s ratio of the four types of bone plates; (**d**) gradient patterns of the density of the four types of bone plates.

The material properties of FGM plates were executed using the user’s subroutines, USDFLD and VUSDFLD, programmed by Fortran 77. In this study, the adoption of USDFLD and VUSDFLD to assign FGM bone plate properties is concise, requiring the definition of only one field variable related to the bone plate coordinates. The carbon fiber volume fraction and the gradient patterns of Young’s modulus, Poisson’s ratio, and the density of the four types of FGM bone plates are shown in Figure 2.

### 2.3. Loading and Boundary Conditions

Osteogenesis capability is influenced by peak principal strain produced by loading modes, which is different when the bone is under compression, bending, and torsion. The peak principal strain produced by compression and bending appeared to be more osteogenic than torsion [52]. Therefore, the bone and fixation plate assembly were modeled under compression and bending, excluding the case of torsion.

In this study, the stress distribution at the fracture site was examined under two distinct loading conditions. The first condition represented the static load experienced during stationary standing, while the second condition simulated the instantaneous load encountered upon contact of the foot with the ground. It was assumed that the magnitudes of these loads were equal, with the difference lying in the mode of loading. Compression was considered as a loading introduced by the patient’s weight when standing up.

**Figure 3:**
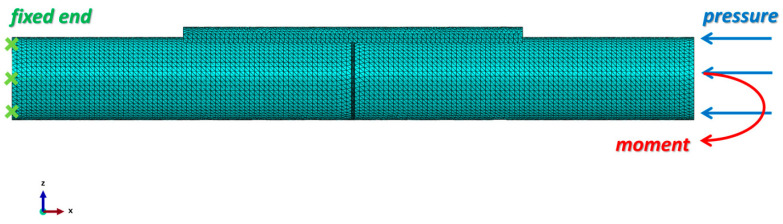
Loading conditions and mesh generation of the fractured bone/plate model. A 10-node quadratic tetrahedron (C3D10) element type was selected for all parts. The left end of the bone was fixed, and at the right end of the bone, the pressure applied was 1 MPa and the moment applied was 1 N·mm. All the screws were tied with the bone fixation plate and the intact bone. The contact property between the intact bone and the fractured bone surfaces was frictionless.

For an 80 kg patient standing with both legs, the approximate load applied to the tibial cross-section can be calculated by (3):

(3) $$p=\frac{F}{A}=\frac{80\times 10}{2\times \pi [(12.5{)}^{2}-5^{2}]}\approx 1.0\;(MPa)$$

where *p* is the mean pressure at the bone cross-section in MPa, *F* is the body weight of the patient in N, and *A* is the cross-section area of the fractured bone site in mm^2^. *A* bending moment of 1 N·mm, which was considered an inducement of callus formation [27], was applied, as shown in Figure 3.

The fractured bone site was arranged to be bonded to the intact bone. For static analysis, simplicity, and the mechanical interaction between the interfaces of the plate and bone surface, the screw and bone were neglected and deliberately considered as fable non-movements or non-interactions; all the screws were tied with the bone fixation plate and the intact bone. Instead, a 0.1 mm gap was predesigned between the fixation plate and the bone surface, as shown in Figure 1.

### 2.4. Finite Element Model

Abaqus 2020 was employed for the simulation. After importing the geometry models from Solidworks 2019, the mesh was generated for all parts. In the static analysis, a 10-node quadratic tetrahedron (C3D10) element type was selected for all parts, while in the dynamic analysis, the element type was C3D10M.

A mesh convergence study was performed to identify the optimal mesh size for the bone and the fixator components. Initially, a coarse element size was selected, and then the mesh size was gradually reduced until the error in the results between the two subsequent steps became less than 2%. The mesh sizes were determined to be 1.5 mm for the intact bone, fixation plate, and screws and 0.5 mm for the fractured bone area. The total number of nodes used in the model is 293,080, and the number of elements is 192,130. The mesh of the assembly is shown in Figure 3. The mesh quality was verified by Abaqus 2020 to ensure the reliability of the simulation results.

## 3. Results and Discussion

### 3.1. Stress Distribution of the Fractured Bone Area under Static Loading

**Figure 4:**
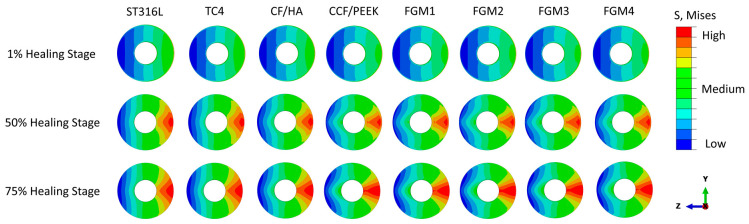
Von Mises stress distributions in the cross-sectional area of fractured bones for different plate materials at the 1%, 50%, and 75% healing stages.

The von Mises stress distributions during the 1%, 50%, and 75% healing stages of the fractured area are presented in Figure 4, illustrating the impact of different bone fixation plate materials. In the examination of the investigated materials, it is evident that the von Mises stress distributions within the fractured bone area are nearly symmetric about the z-x plane, which is expected for symmetrical loading conditions.

However, the von Mises stress increases gradually in the z-y plane from the bone fixation plate side to the opposite side. The minimum stress is on the fixation plate side, which are the expected results, as the fixation plate takes most of the load.

At the early 1% stage of fractured bone healing, which occurs approximately one week after surgery, there is no substantial hard tissue in the fracture gap. Instead, the fractured space is predominately characterized by the presence of hematoma and fluids [5,6,7]. These soft tissues are incapable of bearing mechanical loads because of their relatively small Young’s modulus. As a result, the stresses within the fracture area are relatively low for all bone plate materials, and the stress shielding phenomenon caused by the fixation plate is at the highest level out of the three healing stages.

The von Mises stress distribution at the 50% (three weeks after surgery) and 75% (six weeks after surgery) healing stages within the fractured area presented a similar trend as the 1% healing stage, as shown in Figure 4. The significant difference is the magnitude of the stress.

At the 50% healing stage, the healing tissue becomes a cartilaginous callus that begins to fill in the fracture gap [5,6,7]. Compared with the 1% healing stage, the stress at the fractured bone area increases, as the callus can carry more load. At the 75% healing stage, more calluses are produced, and the calluses become hard enough to carry further external loadings.

**Figure 5:**
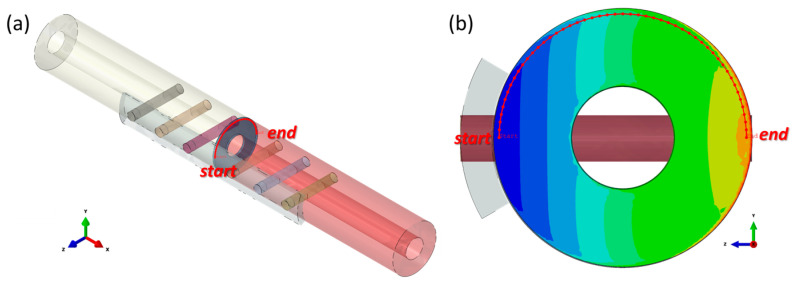
Illustration of the defined path (the red dotted line, r = 11 mm). (**a**) the location of the path in the whole model; (**b**) the location of the path in the fractured bone (the stress contour of the fractured bone is introduced in the corresponding area).

To analyse the stress distribution in the fractured area in detail, a path with a radius of 11 mm (r = 11 mm) along the pitch diameter, as shown in Figure 5, was defined, and the stress on this path has been used to represent the stress distribution characteristics of the fractured area. The path value was determined as the distance travelled along the arc starting from the point below the bone plate on the right side and ending at the point directly across the center.

**Figure 6:**
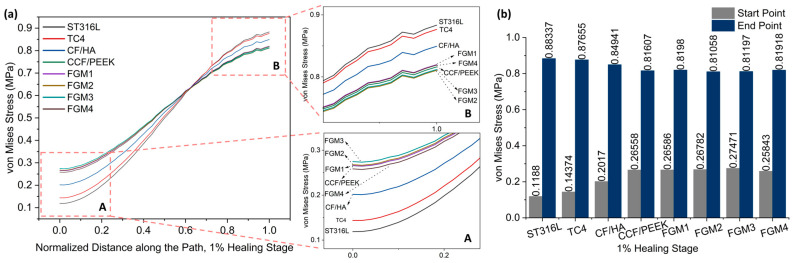
Von Mises stress distributions along the path for the 1% healing stage: (**a**) von Mises stress variation along the path; A and B are magnified views of the start point and end point area; (**b**) a bar chart with specific values of the start point and end point labeled on top of the bar.

**Figure 7:**
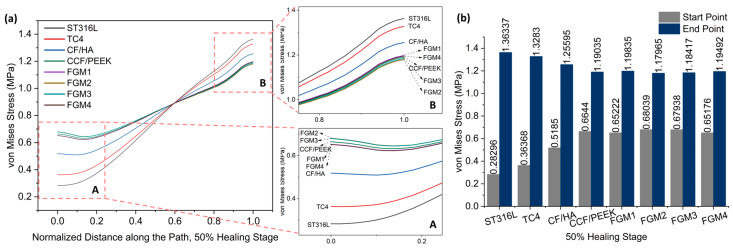
Von Mises stress distributions along the path for the 50% healing stage: (**a**) von Mises stress variation along the path; A and B are magnified views of the start point and end point area; (**b**) a bar chart with specific values of the start point and end point labeled on top of the bar.

**Figure 8:**
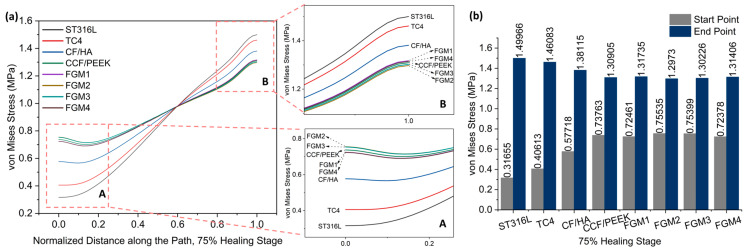
Von Mises stress distributions along the path for the 75% healing stage: (**a**) von Mises stress variation along the path; A and B are magnified views of the start point and end point area; (**b**) a bar chart with specific values of the start point and end point labeled on top of the bar.

Von Mises stresses in a total of 50 nodes on the path were obtained, and the stress distributions along the path at three different healing stages are plotted in Figure 6, Figure 7 and Figure 8. As shown in the figures, different levels of stress shielding were observed at the same fracture site for different fixation plate materials at the same healing stages.

Due to the bone healing beginning at the 1% healing stage, the tissues generated at this stage are not strong enough, and the stress shielding effect is most obvious at this stage. Therefore, it is necessary to pay more attention to this stage.

As shown in Figure 6, it is obvious that the stresses within the fractured area increase gradually from the bone plate side to the areas farther away from it. In Figure 6a, particularly in the magnified view A, it can be observed that during the 1% healing stage, the stresses at the fracture site proximal to the bone fixation plate are the lowest when metallic materials ST316L and TC4 are employed as bone plate materials, followed by the CF/HA bone plate.

In contrast, composite material bone plates can provide greater stress stimulation to the fractured area, which is generally considered beneficial for bone healing. Specifically, FGM3 can provide the highest stress stimulation, followed by FGM2, FGM1, CCF/PEEK, and FGM4. The stress stimulation provided by bone plates made from these four materials is quite comparable, with their stress distribution curves closely overlapping. The stress magnitudes at the start and end points of the path defined in Figure 5 during the 1% healing stage are presented in Figure 6b. It can be observed that at the start point, FGM3 provides the highest stress stimulation, exceeding ST316L, TC4, and CF/HA bone plates by 131.23%, 91.11%, and 36.20%, respectively.

Conversely, as presented in the magnified view in Figure 6a B, in the fractured area that is far away from the bone fixation plate, the stresses are the highest and second highest when metallic materials, ST316L and TC4, are used as bone plates. In contrast, bone plates made of composite materials provide moderate lower stress stimulation compared to metallic plates. Specifically, FGM2 delivers the minimum stress stimulation, followed by FGM3, FGM4, CCF/PEEK, and FGM1. As shown in Figure 6b, FGM2 provides the lowest stress stimulation at the end point. Compared with ST316L, TC4, and CF/HA plates, the stress provided by FGM2 plates was reduced by 8.98%, 8.81%, and 4.79%, respectively. The stress shielding effect is more serious in the area close to the bone plate (start point in Figure 5), and the selection of bone plate materials has a significantly greater impact on the stress shielding effect in the area near the bone plate than the area far away from the bone plate. Therefore, when selecting bone plate materials, priority should be given to addressing the stress shielding effects near the bone plate over those at locations farther from the bone plate.

Figure 7 and Figure 8 present stress variation along the defined path, as shown in Figure 5, during the 50% and 75% healing stages, respectively. It can be seen that the stresses of these two stages change in exactly the same trend. Different from the 1% healing stage, the maximum stress at the start point at the 50% and 75% healing stages were both provided by FGM2. However, the magnitude of stress provided by FGM3 and FGM2 is very close, with a difference of less than 0.2% in both stages. The minimum stress at the start point and the maximum stress at the end point were still provided by ST316L, and are the same as in the 1% stage. In the 50% and 75% healing stages, the stress provided by FGM2 at the start point was 140.45% and 138.62 higher than ST631L, respectively. FGM3, which had the second highest stress at the start point, can still provide 140.10% and 138.19% higher stress than ST316L, respectively.

According to the stress at the start point and end point in the three healing stages, the FGM3 bone plate can provide the highest stress to the fractured area. However, although FGM3 provides the maximum stress (1% healing stage) and second maximum stress (50% and 75% healing stages) at the start point, it has the second lowest stress at the end point at all healing stages. Although the analysis above concluded that the stress at the end point was not as sensitive to the selection of the materials as the start point, in order to better determine whether FGM3 is the most suitable bone plate material, the average stresses of the 50 nodes on the path we defined in Figure 5 were calculated to observe the stress shielding effects.

**Figure 9:**
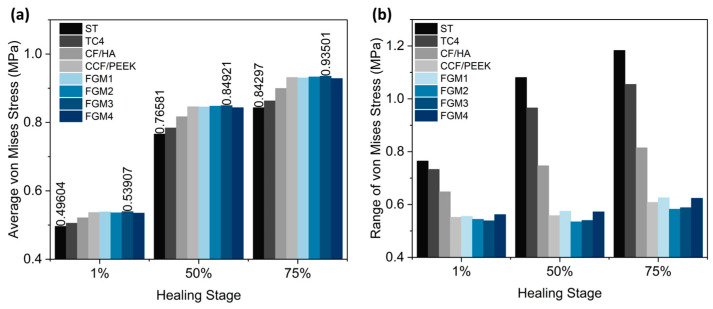
The average von Mises stress and ranges in the fractured area for different material fixation plates and healing stages: (**a**) the average von Mises stress; the values of the maximum and minimum stresses are labeled on the top of the bar; (**b**) the range of von Mises stress.

The average von Mises stress and ranges in the fractured area using different material fixation plates at different healing stages were plotted in Figure 9a,b, respectively, and the maximum and minimum stress were labeled on top of the bar in Figure 9a. As illustrated in Figure 9a, the FGM3 bone plate provides the highest average stress at all three healing stages, while ST316L provides the lowest average stress. Compared with ST316L, the average stresses provided by the FGM3 plate are, respectively, 8.67%, 10.89%, and 10.92% higher in the healing stages of 1%, 50%, and 75%. At the same time, the ranges of the stress for the FGM3 plate are the lowest (1% healing stage) and second lowest (50% and 75% healing stages) out of all types of plate materials, as shown in Figure 9b. It is clear that an FGM3 bone plate can introduce the highest mean stress to the fractured area and ensure the stress variation in the fractured area is relatively small at the same time.

The results above demonstrated that FGM3 bone fixation plates have the smallest stress shielding compared with other fixation plates at all three healing stages investigated. The decreasing tendency of the stress shielding along the path was similar among the four different bone fixation plates, showing that the FGM3 bone plate proved to provide the best healing performance compared to the other types of bone fixation plates.

### 3.2. Stress Distribution of the Bone Fixation Plates under Static Loading

**Figure 10:**
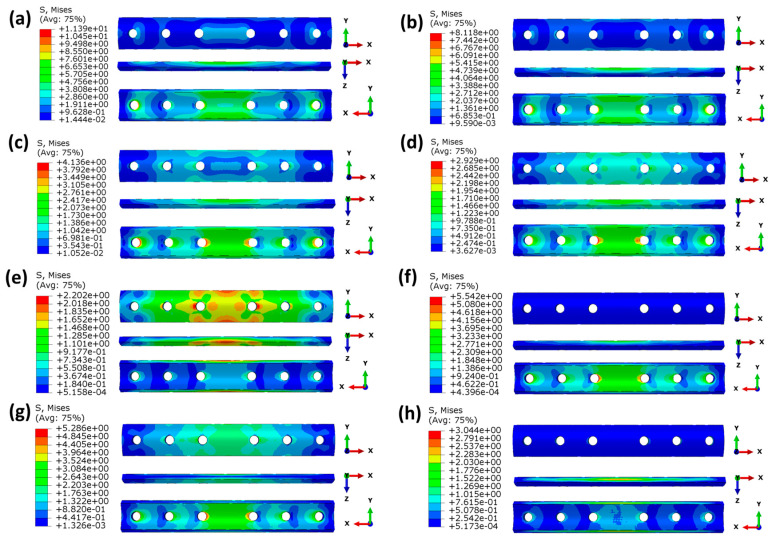
Von Mises stress (MPa) distributions of the plates at the 1% healing stage: (**a**) ST316L; (**b**) TC4; (**c**) CF/HA; (**d**)CCF/PEEK; (**e**) FGM1; (**f**) FGM2; (**g**) FGM3; (**h**) FGM4.

In terms of mechanics, analyzing the stress distribution within the bone plate, which serves as a structural part to support the fractured area, is an important method to investigate the mechanical behavior of bone plates. At the 1% healing stage, the fractured area is soft and weak and has the lowest capacity to bear external loads. The majority of the load is borne by the bone plate, leading to the highest stress within the plate at this stage. Therefore, fixation plates at the 1% healing stage were selected for analysis, as shown in Figure 10.

As shown in Figure 10a–d, the maximum stresses of bone plates made of homogeneous materials gradually decrease with a decrease in the material’s modulus because bone plates with a higher modulus bear greater stress. The stress concentration is mainly at the edge of the screw hole, and the stress concentration is the largest in the middle two screw holes near the fractured site. This is because the fractured area is too weak to withstand the external stress, and most of the stress is transferred by the bone plate and screw. Thus, the stress will be concentrated in the screw hole edge. As shown in the X-Y perspective in Figure 10a–d, the stress in homogeneous bone plates decreases gradually from the inner surface to the outer surface. This is because the bone plates designed in this paper are concentric fan-shaped, and the volume increases with an increase in the radius.

As can be seen in Figure 10e–h, for the four FGM bone plates, although the average modulus is the same as CCF/PEEK, the stress distribution in the FGM bone plates is significantly different from the CCF/PEEK bone plate. This is because of the different distribution of the elastic modulus in the FGM bone. As the inner surface of FGM2 and FGM3 bone plates has a greater elastic modulus, and the stress value of the inner surface itself is greater than the outer surface, according to the analysis of homogeneous bone plates mentioned above, and the maximum stress values of FGM2 and FGM3 are much higher than CF/PEEK, FGM1, and FGM4.

### 3.3. Stress Distribution of the Fractured Area under Dynamic Loading

Research by Bawiskar [53] found that early rehabilitation training has a positive impact on patients’ long-term functional recovery, postoperative bone strength, postoperative confidence, and gait improvement. In the case of a patient with a fractured tibia standing on both legs, the load on the legs can be regarded as stable and evenly distributed. However, during early rehabilitation training processes, such as walking, the stress stimulus on the fractured area at the moment of foot contact with the ground differs from the static condition. Therefore, a simulation of the stress distribution at the fracture site under dynamic loading conditions was conducted.

Within the first week after fracture surgery, the fracture site requires maximum protection. Thus, walking exercises are not performed during this stage. Walking rehabilitation training is typically started during the mid and late stages (50% and 75% healing stages) of fracture recovery.

Assuming that during the walking rehabilitation training process, the magnitude and direction of external forces acting on the fracture site are equivalent to those experienced when the patient is standing on both feet. The difference lies in the loading pattern of the external forces, which is instantaneous. Under this circumstance, the stress distribution at the fracture site changes over time. This simulation aimed to investigate the stress distribution during dynamic activities and their influence on the healing process.

**Figure 11:**
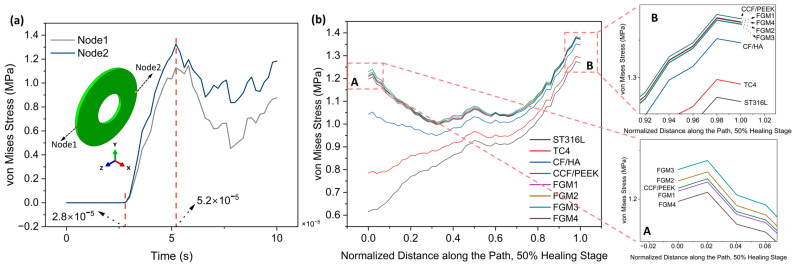
Von Mises stress distributions for the 50% healing stage: (**a**) the variation of von Mises stress with the time of node 1 and node 2 (both nodes are located on the edge of the fractured site); (**b**) von Mises stress distributions along the path for the 50% healing stage; A and B are magnified views of the start point and end point area.

In this section, the 50% healing stage was selected for analysis. Different from static analysis, the stress in dynamic analysis changes with time. To evaluate the stress shielding effect in dynamic analysis, two nodes, labeled node 1 and node 2, located on the edge of the fractured site, as shown in Figure 11a, were selected to monitor stress variations. The result in Figure 11a reveals that before the time of 2.8 × 10^−5^ s, the stress values are 0; this is because the stress wave has not yet propagated to the fractured site. After the time of 2.8 × 10^−5^ s, the stresses on the two nodes gradually increase and reach the maximum values at the time of 5.2 × 10^−5^ s, and subsequently decrease as the stress wave passes through the selected nodes. To compare the stress values under instantaneous loading conditions for different materials, a path was defined in the static analysis (Figure 5), with 50 nodes on the path. Stress data from 50 nodes of different bone plates along this path were collected at a time of 5.2 × 10^−5^ s, and the results are shown in Figure 11b.

Compared with the results of static loading (Figure 7), the results of instantaneous loading are significantly different. The stresses provided by ST316L and TC4 plates within the fractured area increase gradually from the start point to end point, whereas for stress provided by other plate materials, stresses decrease first and then increase again. At the start point, both CCF/PPEK plates and FGM plates possess significantly higher stress values compared to CF/HA, TC4, and ST316L plates, which are similar than static loading conditions. As shown in Figure 11b A, FGM3 can provide the highest stress stimulation at the start point, followed by FGM2, CCF/PEEK, FGM1, and FGM4.

Similarly, as presented in the magnified view in Figure 11b B, at the end point, bone plates made of composite materials provide higher stress stimulation compared to metallic plates. Specifically, the CCF/PEEK plate provides the maximum stress stimulation, followed by FGM1, FGM4, FGM2, and FGM3. In contrast, the stresses are the lowest and second lowest when metallic materials ST316L and TC4 are used as bone plates.

As mentioned in the previous section, compared with the end point, the stress shielding effect is more serious at the start point due to the start point being closer to the bone plate. Under instantaneous loading, the maximum stress at the starting point is observed when the FGM3 bone plate is used, indicating that it is the preferred choice among all the bone plates.

**Figure 12:**
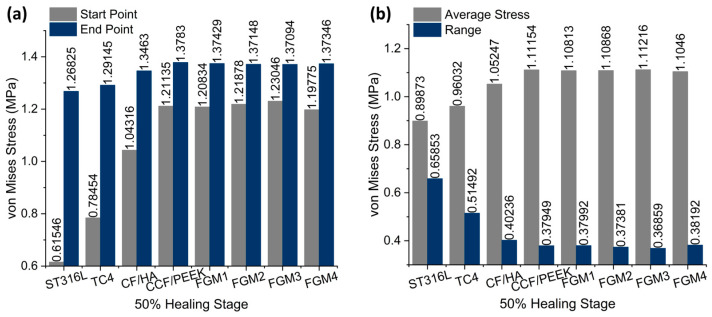
The average von Mises stress and ranges in the fractured area for different material fixation plates and healing stages: (**a**) the average von Mises stress; the values are labeled on the top of the bar; (**b**) the range of von Mises stress; the values are labeled on the top of the bar.

As shown in Figure 12a, at the start point, FGM3 provides the highest stress stimulation, exceeding ST316L, TC4, and CF/HA bone plates by 99.93%, 56.84%, and 17.96% respectively. Furthermore, at the end point of the fractured area, FGM3 can still provide 8.10% more stress than the ST316L plate. Under static loading, the ST316L plate can provide 15.13% more stress than the FGM3 plate at the end point.

Similar to the static analysis, calculations for the average and range of all points along the path were performed, as shown in Figure 12b. The FGM3 bone plate provides the highest average stress at the 50% healing stage, while ST316L provides the lowest average stress, as shown in Figure 12b. Compared with ST316L, the average stress provided by the FGM3 plate is 23.75% higher. Moreover, the range of the von Mises stress for FGM3 is the lowest among all types of plate materials. It is clear that under instantaneous loading, the FGM3 bone plate can also introduce the highest mean stress to the fractured area and ensure that the stress variation in the fractured area is lowest. However, the FGM bone plate models were defined using ABAQUS and the user’s subroutines, USDFLD and VUSDFLD. Due to the difficulty of determining the interaction between the bone and plate, the frictional contact between the bone and plate makes it impossible to introduce frictional contact modes in this work. Therefore, the completed contact mode is adopted. In addition, the effect of poroelasticity on the bone is not included in this investigation.

## 4. Conclusions

Finite element analysis was employed in this study to simulate the influence of different types of chopped carbon fiber-reinforced PEEK (polyether ether ketone) functionally graded composite materials for bone fixation plates on the stress shielding effects at the fractured site under static and instantaneous dynamic loading conditions. Comparisons were made in this study against traditional bone repair plate materials, such as ST316L and TC4. The simulation results revealed that under both static and instantaneous loading, chopped carbon fiber-reinforced PEEK bone plates (both homogenous and functionally graded materials) could provide low stress shielding performance in a higher stress stimulation scenario compared to traditional metallic bone plates. This was evident not only in the stress at the fractured site close to the bone plate but also in the overall average stress across the fractured bone site.

Within the bone plates made of functionally graded materials and despite the fact that all FGM bone plates have the same average elastic modulus, FGM3 (with an elastic modulus gradually increasing from the center to both sides) had the best performance. Under static and instantaneous dynamic loading, FGM3 not only had the highest stress at the fractured site close to the bone plate but also the maximum average stress across the entire fractured bone area. Moreover, FGM3 provided the most uniform stress distribution.

Based on the physical and chemical characteristics required for fracture healing, this work suggests that the FGM3 fixation plate is the most suitable material for the fracture healing process. This is because FGM3 facilitates better stress distribution within the fractured area. In comparison to metallic bone fixation plates currently used in biomedical and healthcare applications, according to our numerical modeling and dynamic simulation, the FGM3 bone fixation plate is more suitable for internal bone fixation applications.

## Figures and Tables

**Table 1:** The mechanical properties of all materials used in the modeling analysis [10,23,27,45,46,47,48,49].

Material	E_x_ (MPa)	E_y_ (MPa)	E_z_ (MPa)	nu_xy_	nu_yz_	nu_xz_	Gxy (MPa)	Gyz (MPa)	Gxz (MPa)	ρ (g/cm^3^)
ST316L	210,000	210,000	210,000	0.300						8.000
TC4	110,000	110,000	110,000	0.300						4.430
CF/HA	78,000	47,900	47,900	0.270			5800	5800	5800	2.480
PEEK	4000	4000	4000	0.390						1.300
Carbon Fiber	231,000	231,000	231,000	0.270						1.790
CCF/PEEK	22,530	22,530	22,530	0.440						1.072
Bone	18,400	6910	8510	0.488	0.307	0.315	2410	3560	3560	1.916
Fractured Bone	200 (1% healing)				0.300					0.731
	10,000 (50% healing)				0.300					1.434
	15,000 (75% healing)				0.300					1.699

## Data Availability

The ABAQUS user’s subroutines, USDFLD and VUSDFLD, mentioned in this study are available from GitHub (https://github.com/DemonMelon/USDFLD-VUSDFLD (accessed on 13 January 2024)).
